# Setup errors in patients with head-neck cancer (HNC), treated using the Intensity Modulated Radiation Therapy (IMRT) technique: how it influences the customised immobilisation systems, patient’s pain and anxiety

**DOI:** 10.1186/s13014-017-0807-y

**Published:** 2017-04-27

**Authors:** Massimiliano Contesini, Monica Guberti, Roberta Saccani, Luca Braglia, Cinzia Iotti, Andrea Botti, Emilio Abbati, Marina Iemmi

**Affiliations:** 1 Human Resource Development – Training, Azienda Unità Sanitaria Locale, Reggio Emilia, Italy; 2 Nursing Directorate, Arcispedale Santa Maria Nuova-IRCCS, Reggio Emilia, Italy; 3 Radiation Oncology Department, Arcispedale S. Maria Nuova –IRCCS, Reggio Emilia, Italy; 4 Scientific Directorate, Arcispedale S. Maria Nuova – IRCCS, Reggio Emilia, Italy; 5 Medical Physics Department, Arcispedale S.Maria Nuova –IRCCS, Reggio Emilia, Italy

**Keywords:** HNC, IMRT, Setup error, Pain, Anxiety, Customised immobilisation systems

## Abstract

**Background:**

In patients with head-neck cancer treated with IMRT, immobility of the upper part of the body during radiation is maintained by means of customised immobilisation devices.

The main purpose of this study was to determine how the procedures for preparation of customised immobilisation systems and the patients characteristics influence the extent of setup errors.

**Methods:**

A longitudinal, prospective study involving 29 patients treated with IMRT. Data were collected before CT simulation and during all the treatment sessions (528 setup errors analysed overall); the correlation with possible risk factors for setup errors was explored using a linear mixed model.

**Results:**

Setup errors were not influenced by the patient’s anxiety and pain. Temporary removal of the thermoplastic mask before carrying out the CT simulation shows statistically borderline, clinically relevant, increase of setup errors (+24.7%, 95% CI: −0.5% - 55.8%). Moreover, a unit increase of radiation therapists who model the customised thermoplastic mask is associated to a −18% (−29.2% - -4.9%) reduction of the errors.

The setup error is influenced by the patient’s physical features; in particular, it increases both in patients in whom the treatment position is obtained with ‘Shoulder down’ (+27.9%, 2.2% - 59.7%) and in patients with ‘Scoliosis/kyphosis’ problems (+65.4%, 2.3% - 164.2%). Using a ‘Small size standard plus customized neck support device’ is associated to a −52.3% (−73.7% - -11.2%) reduction.

The increase in number of radiation therapists encountered during the entire treatment cycle does not show associations. Increase in the body mass index is associated with a slight reduction in setup error by (−2.8%, −5% - -0.7%).

**Conclusion:**

The position of the patient obtained by forcing the shoulders downwards, clinically significant scoliosis or kyphosis and the reduction of the number of radiation therapists who model the thermoplastic mask are found to be statistically significant risk factors that can cause an increase in setup errors, while the use of ‘Small size’ neck support device and patient BMI can diminish them.

## Introduction

Intensity modulated radiation therapy is the standard for the treatment of head-neck cancer [1], because it allows a distribution in high doses in conformity to the tumour, saving the adjacent normal structures.

IMRT generates high gradient doses on the target, with rapid drop in the latter to the level of normal structures; as a result, this technique requires extremely high precision in treatment; in fact, very minor positioning errors can affect the target cover and increase the dose to organs at risk (OARs) [2, 3].

Image guided radiation therapy (IGRT) techniques have been developed and used to ensure accurate positioning of the interfraction patient and obtain a repeatable dispensing of the dose [4–7].

In patients with head-neck cancer (HNC) treated with IMRT, immobility of the upper part of the body during radiation is usually maintained by means of customised immobilisation devices such as head-neck-shoulder thermoplastic masks [8, 9]. The main purpose of this study was to explore how patients characteristics, the procedures for preparation of customised immobilisation systems and their subsequent use can influence the accuracy and reproducibility of treatment.

## Methods

### Study design and assessment procedure

This is a longitudinal, prospective study: data were collected before CT simulation and during all the treatment sessions. All the patients enrolled were identified by a numeric code to allow blind data analysis and ensure the patient’s privacy. Written informed consent was obtained during the first radiation therapy visit. The information was provided by the same radiation therapists for all the patients involved in the study, in order to ensure homogeneity. The local Ethics Committee approved the study.

### Setting

In our department radiation therapy treatments can be performed using the TomoTherapy® Hi-Art® System 3.0 (Accuray Incorporated, Madison, WI), which involves a helical fan beam scan using 3.5MV photons and an arc-shaped xenon CT detector array mounted on the opposite side of the ring gantry Mega Volt Fan Beam Computed Tomography (MVFBCT).

All patients with head-neck tumour were immobilised using a 5-points mask: four fixing points for the neck and shoulders plus an additional one on the head (Acquaplast RT® Fibreplast ™ Thermoplastics, Avondale, PA, USA). The patient’s position was maintained by means of a standard rigid base made of carbon fibre, created specially to stabilise the patient’s head and neck during IMRT treatments. This supporting base of the patient is always fixed to the Computer Tomography and the Tomotherapy beds.

To maintain the patient’s position standard neck supports were used in different sizes (Large, Small size), connected to the base. In some cases, to improve patient comfort, an additional customised neck support was used (MOLDCARE® CUSHION For Head, Neck and Small Regions Rt -4492S, ALCARE Co., Ltd. Tokyo, Japan). All the patients enrolled were subjected to Virtual Simulation Computed Tomography (GE Medical System HiSPEED NX/I) using the same technical protocol (120 kV, 250 mA, Thickness of acquisition 3 mm, Pitch 3, Thickness of reconstruction 3 mm, Reconstruction interval 3 mm).

Before each treatment, patient positioning was obtained by means of alignment of the lasers of the room with 4 reference points positioned on the “mask” at the time of CT simulation.

In IGRT-IMRT treatments dispensed in Tomotherapy, the MVFBCT images were acquired daily before each treatment and matched with those obtained during the CT simulation. Alignment of the images was obtained by means of automatic software tools and, if necessary, also with manual adjustments [10–12].

We considered the translational mediolateral (ML), craniocaudal (CC), and anteroposterior (AP) deviations; antero-posterior correction of 3.8 mm is applied for the systematic AP setup error caused by couch sag [13–16] that occurs when the patient is moved to the treatment isocenter (inside the bore) from virtual isocenter located 70 cm outside. Rotational corrections like pitch, roll and yaw were not recorded.

### Patients

We prospectively collected data for a sample of consecutive patients with head and neck cancer, treated with the IMRT technique by our department from September 2011 to August 2013.

The eligibility criteria were: female and male patients with head and neck cancer who underwent intensity modulated radiation therapy, ≥14 years old and ≤85 years old, without cognitive disfunctions, performance status score of 0–1 [17] and with written informed consent.

### Data collection

For all the patients, the thermoplastic mask and other custom immobilization devices were prepared before CT simulation, on the basis of the same internal operating protocol.

We created an ad-hoc report to record all the environmental and technical variables that might have a correlation with the setup error. On the baseline (BL) day of CT simulation we recorded: anxiety [18] and pain [19–24], before starting the simulation procedure (more on the measurements scales below); patient’s weight and height [25, 26]; intent of radiation therapy; variable influencing patient positioning such as shoulders forced down, scoliosis or kyphosis; some patient and environment variables such as presence of mobile dental prosthesis, use of bite for locking of the jaw, tracheostomy, voluminous beard, voluminous hair, room temperature of the CT room and temperature of the water used for shaping the thermoplastic mask; type of standard neck supports used and the eventual addition of customized neck support; number of radiation therapy team members who worked together to shape the thermoplastic mask and temporary removal of the mask before the acquisition of CT simulation. On the first day of every week of treatment we measured the patient’s weight. Finally on each treatment day we recorded: Mediolateral (ML), craniocaudal (CC), and anteroposterior (AP) deviations; radiation therapists executing the procedure; anxiety and pain, before starting the treatment session; changes related to the presence of mobile dental prosthesis, use of dental bite, tracheostomy, beard or voluminous hair, temperature of the treatment room.

The anxiety was measured using the STAI Y form, a test validated worldwide [27] consisting of two scales: the TRATTO-A scale, which measures the predisposition for anxiety, and the STATE-A scale which evaluates the anxiety state, i.e. the emotional state at a given moment. Only STATE-A was used in the study, which consists of 20 items; the compilation, done directly by the patient, requires an average of 5–7 min. The test was administered to the patient in the waiting room.

Pain was measured using the Numeric Pain Rating Scale, [19–24] a numeric scale that describes the intensity of pain, ranging from 0 (no pain) to 10 (max value of pain); the measurement was performed directly by the patients themselves in the waiting rooms.

### Study size

In absence of a-priori hypothesis given the exploratory nature of the study, no formal sample sizing was performed. Nonetheless, a limited measurement period (recruiting: September 2011 to August 2013, data collection: first 6 weeks of treatment for each patient) was defined, in order to ensure general feasibility, homogeneity of the procedures and of the data collected.

### Statistical methods

Clinical and demographic data were expressed in terms of frequency and percentage for categorical variables, mean ± standard deviation for symmetric quantitative variables, median + IQR for skewed ones.

Overall mean error (*M*), systematic SD (*∑*) and random SD (*σ*) were calculated, both for full sample and by groups (determined according to site) of patients.

In order to explore the correlation between relevant variable and treatment setup errors, we adopted a single Euclidean distance measure. Setup error (in any of the three directions) can assume positive or negative values; regardless of the direction, the more the error deviates from value 0 the more unfavourable the it is. Therefore, to collapse all the three errors (cranial-caudal, medial-lateral and anterior-posterior) in one single measurement readily available for multivariate analysis, we considered each setup error as a vector and calculated 3-dimension modulus as an overall measure of error severity; accordingly, total error *e*_*tot*_ was simply calculated as Euclidean distance, as follows:

$$ {e}_{tot}=\sqrt{e_{ml}^2+{e}_{cc}^2+{e}_{ap}^2} $$

where *e*_*ml*_ is medial-lateral (ML), *e*_*cc*_ is cranial-caudal (CC) and *e*_*ap*_ is anterior-posterior (AP) error (to simplify notation we avoided patient and time subscripting; however *e*_*tot*_ has to be considered as overall setup error for patient *i* at time *t*). The total error, expressed in mm, was log-transformed (in order to mitigate the positive-skewness effects on the residuals normality) and then analysed using a linear mixed effects model (complete-case analysis). This method was choosed to accommodate non-independency of the observations sampled (eg. due to clustering within individuals): the method assumes that setup errors can be explained in terms of both fixed and random effects. Fixed effects represent the effects of factors of intrinsic interest (in our case those that can be associated to setup error) because of being repeatable in other populations (eg number of therapists, mask removal etc.); random effects represent random deviation (from the relation depicted by the fixed effects) associated to factors of no intrinsic interest (not strictly repeatable, eg the single patient) which are nonetheless considered in the estimation process [28].

Covariates used in the model were suggested by clinical/technical interest and feasibility (some patient characteristic, eg voluminous beard etc., were highly imbalanced and therefore were ignored for the correlation analysis): fixed effect variables were shoulders down, scoliosis/kyphosis and mask removal (No/Yes variables), type of neck support device (categorical variable), number of radiation therapists involved at baseline, pain, anxiety, number of radiation therapists encountered during the treatment weeks and body mass index (continuous variables treated as such also in the statistical model); a random patient intercept complete the model presented. We compared this model with one including some additional variables (fixed effects: pain and anxiety before each treatment session, both hair and temperature at baseline and before each treatment session; random intercept: treatment technician), concluding that these latter were not needed (*P* = 0.98).

The base group for the model reported is represented by patients without ‘Shoulders down’, ‘Scoliosis/kyphosis’ and ‘Mask removal’ at baseline setup; with ‘Large size standard plus customized neck support device’ (the most common); followed by 2 radiation therapists at baseline for mask and setup (the minimum/standard encountered); with no pain (pain = 0); with mid-point anxiety (anxiety = 50); patient who during the entire treatment cycle, was followed by 12 radiation therapists (minimum) during treatment weeks; with a mean BMI.

In order to ease interpretation of results, coefficients (and their confidence intervals) were transformed using (((exp(coefficient) – 1)*100)-100) and presented using a forestplot-like graph: this leads to an estimate of each factor contribution as percentage variation (compared to an otherwise identical patients without it).

Confidence intervals (considering a 0.95 confidence level) and models comparison were performed with bootstrap methods [29]; a confidence interval not including the threshold of no differences (0% for transformed coefficients) denoted the contribution of the factor analysed as statistically significant. Statistical analysis was carried out using R 3.3.2.

## Results

### Patients

The number of eligible patients screened at our radiation therapy centre for IMRT treatment from September 2011 to August 2013 was thirty-nine (39); seven (7) patients did not give their consent for research; two (2) patients were discontinued from the study since their treatment had to be re-planned and the mask had to be remade as it had lost its accuracy during the radiation therapy cycle; one patient (1) dropped out because of increased pain and poor collaboration. Therefore the analysed sample consisted of twenty-nine (29) consecutive patients (for a total of 528 daily images); descriptive statistics of the patients at baseline (CT Simulation) are shown in (Table 1).

**Table 1:** Baseline descriptive statistics (CT simulation)

Patients, n %		29	100
Gender, n %			
	Female	9	31.0%
	Male	20	69.0%
Age, Median (IQR)		65.7	(58.9–71.9)
Site, n %			
	Nasopharynx	5	17.2%
	Oropharynx	13	44.8%
	Hypopharynx	5	17.2%
	Oral cavity	4	13.8%
	Other	2	6.9%
Setting, n %			
	Radical	23	79.3%
	Post surgery	6	20.7%
Shoulders down, n %			
	No	18	62.1%
	Yes	10	34.5%
	Not available	1	3.4%
Scoliosis/kyphosis, n %			
	No	26	89.7%
	Yes	3	10.3%
BMI, mean (sd)		24.07	(4.34)
Mask removal, n %			
	No	13	44.8%
	Yes	15	51.7%
	Not available	1	3.4%
Neck support device, n %			
	Large size standard plus customized neck support device	23	79.3%
	Large size standard	4	13.8%
	Small size standard plus customized neck support device	2	6.9%
Baseline radiation therapists, n %			
	1	2	6.9%
	2	23	79.3%
	3	3	10.3%
	4	1	3.4%
Pain, Median (IQR)		0	(0–2)
Anxiety, Median (IQR)		42	(36–64)

### Outcome descriptives

Overall mean error (*M*), systematic error (*∑*) and random error (*σ*) are shown in (Table 2).

**Table 2:** Population setup errors

		Tomotherapy		
		M	Σ	σ
All patients				
	Mediolateral	−0.4	3.1	2.2
	Craniocaudal	2.0	1.6	1.7
	Anteroposterior^a^	0.8	1.3	1.7
Main sites:				
Nasopharynx				
	Mediolateral	1.5	2.8	2.6
	Craniocaudal	1.2	1.1	2.5
	Anteroposterior^a^	0.7	1.8	1.5
Oropharynx				
	Mediolateral	−1.6	2.9	2.5
	Craniocaudal	1.8	1.4	1.6
	Anteroposterior^a^	0.7	1.4	1.9
Hypopharynx				
	Mediolateral	−0.3	3.4	1.7
	Craniocaudal	2.6	1.7	1.6
	Anteroposterior^a^	1.1	0.9	1.3
Oral cavity				
	Mediolateral	1.2	1.5	1.4
	Craniocaudal	2.0	1.4	1.3
	Anteroposterior^a^	0.5	1.2	1.6
Other				
	Mediolateral	2.0	3.3	1.9
	Craniocaudal	4.6	3.0	1.5
	Anteroposterior^a^	1.7	1.5	1.3

^a^Antero-Posterior correction of 3.8 mm is applied for the systematic AP setup error caused by couch sag

The mean displacements in ML, CC and AP were −0.4, 2.0 and 0.8 mm. Systematic and random errors were 3.1, 1.6, 1.3 and 2.2, 1.7, 1.7 in the ML, CC and AP directions, respectively.

### Main results

**Fig. 1:**
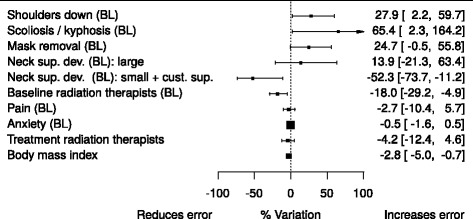
Model results: setup error variation associated to each factor analysed

The mixed effects model is presented in (Table 3) while a graphical summary of results is plotted in (Fig. 1).

**Table 3:** A linear mixed effect model: fixed effect coefficients

	Estimate	Std. Error	95% CI	
			Lower limit	Upper limit
Intercept	1.327	0.099	1.137	1.518
Shoulders down (BL) (*)	0.246	0.115	0.022	0.468
Scoliosis/kyphosis (BL)	0.503	0.244	0.023	0.972
Mask removal (BL)	0.221	0.114	−0.005	0.444
Neck support device (BL):				
Large size, standard	0.130	0.185	−0.240	0.491
Small size, custom	−0.740	0.309	−1.337	−0.119
Baseline technicians (BL)	−0.198	0.074	−0.346	−0.051
Pain (BL)	−0.027	0.042	−0.110	0.055
Anxiety (BL)	−0.005	0.005	−0.016	0.005
Treament technicians	−0.043	0.045	−0.132	0.045
Body mass index	−0.029	0.011	−0.051	−0.007

(*) ‘BL’ means a baseline measured variable

Considering the graph, factors associated with a diminished setup error have a confidence interval on the left of the vertical line (negative percent variation), while others with confidence interval on the right are associated to an increased setup error: the percentage variation is estimated by referencing to a patient identical in every aspect but the risk factor considered in turn.

Having *‘*Shoulders down’ and *‘*Scoliosis/kyphosis’ is associated to a 27.9% (95% CI: 2.2% - 59.7%) and 65.4% (2.3% - 164.2%) increase in mean setup error respectively. Mask removal is associated to a (borderline) non significant increase in setup errors (+24.7%, −0.5% - 55.8%). Using a ‘Small size standard plus customized neck support device’ is associated with a −52.3% (−73.7%, −11.2%) setup error contraction; less clear indications are available on the use of customized neck support (base group) in patients with ‘Large size standard’ neck support device.

No significant relations were found between the number of radiation therapists encountered during the entire treatment schedule and setup errors; on the other hand, a unit increase in the number of radiation therapists involved in CT Simulation for making the mask is associated with a −18% (−29.2% - -4.9%) decrease of setup errors.

Increased BMI coefficient suggests a mild protective effect, −2.8% (−5%, −0.7%) per unit; ., no associations were found for anxiety and pain.

## Discussion

### Key results and limitations

This study was a mono-institutional experience, conducted in an explorative way without a formal process of study dimensioning, aimed at exploring the correlation between setup error and possible risk factors. First, statistically significant results were obtained for Scoliosis/kyphosis; shoulders down was, as expected, predictive of clinically relevant increase of set-up error. These results may be explained by the fact that such anatomical conditions of the patient are associated with less comfort and result in lower reproducibility of the position. The use of a *‘*Small size standard plus customized neck support device’ is associated with reduction in set-up errors; this is probably due to the fact that such an immobilization system ensures a greater restraint of the patient’s head and neck.

The unit increase in the number of radiation therapists at baseline is associated with an overall reduction of average setup errors; this can be explained by the fact that increase in the number of radiation therapists involved in shaping the thermoplastic mask on the patient ensures greater accuracy in the preparation of the patient immobilisation system.

The results of the study show that temporary removal of the mask before CT simulation acquisition is associated with a clinically significant increases of the setup error, despite the association in not strictly statistical significant; this provides clues on the probable usefulness of immobilisation procedure in claustrophobic patients (in whom it is often necessary to remove the immobilisation system before the CT simulation).

The patient’s pain and anxiety measured in the CT simulation phase were not associated with a clinically significant increase of setup errors. These results can be explained by the fact that the members of the radiation therapy team received training in communication strategies which may be used to reduce patient anxiety. Patient pain, despite increasing gradually during the radiation therapy cycle, was well controlled with pharmacological treatment.

The number of radiation therapists encountered during the treatment schedule was not associated with clinically relevant increase set-up error; this can be explained by the fact that the high standardization of treatment procedures ensures the reproducibility of the patient setup, which is not operator dependent. The results also showed that factors such as voluminous hair, room temperature and temperature of the water used for shaping the thermoplastic mask were not associated with SE.

To the best of our knowledge, this is the first study to prospectively investigate the relations between SE and the procedures for preparation of customized immobilization systems: however, these findings need to be confirmed. We can highlight as a point of weakness that geometric corrections such as pitch, roll, and yaw were not considered; moreover, we also need to report the limited numbers of patients.

## Conclusion

Factor associated with an increased error were ‘Shoulders down’ and scoliosis/kyphosis; factor associated with a diminished error were the use of small size plus custom neck support device, the number of radiation therapists involved in shaping the thermoplastic mask during CT Simulation and BMI.

The results of the study were obtained from a small sample size and need to be confirmed, but provide suggestions useful to the radiation therapist in the treatment preparation phase and in the positioning of the patient daily during therapy.

## Abbreviations

AP: Anteroposterior
BMI: Body mass index
CC: Craniocaudal
DRRs: Digitally reconstructed radiography
EPID: Electronic Portal Imaging Device
HNC: Head-neck cancer
IGRT: Image guided radiation therapy
IMRT: Intensity modulated radiation therapy
ML: mediolateral
MVFBCT: Mega Volt Fan Beam Computed Tomography
OARs: Organs at risk
SE: Setup errors
